# Language Learning Activities in Educational Settings Incrementally Predict Language Ability Beyond Daily Conversation in Preschoolers with Cochlear Implants: A Comparative Study with Normal-Hearing Peers

**DOI:** 10.3390/children13060806

**Published:** 2026-06-11

**Authors:** Meilin He, Chie Obuchi, Inho Chung

**Affiliations:** 1 Doctoral Program in Disability Sciences, Graduate School of Comprehensive Human Sciences, University of Tsukuba, Ibaraki 305-8572, Japan; 2 Institute of Human Sciences, University of Tsukuba, Ibaraki 305-8572, Japan; ichungjp@gmail.com

**Keywords:** hearing-impaired, preschool children with cochlear implants, language activities, educational settings, language ability

## Abstract

Background/Objectives: This study examined how different types of language activities in educational settings predict language ability in preschoolers with cochlear implants (CIs) versus normal-hearing (NH) peers. Predictor variables included two dimensions of language activities (language learning activities: shared reading, word games, and story retelling; daily communication activities: routine conversations and free play talk), as well as auditory utilization status (awareness of sounds, perception of speech sounds, and attentive listening to teachers/peers), learning attentional state, and family socioeconomic status (SERS). The outcome variable was language ability, measured with the Mandarin Clinical Evaluation of Language for Preschoolers’ Core Scale (MCELP-CS), and control variables included age and, for the CI group only, age of implantation. Methods: Participants included 189 CI (mean age = 4.53 years, SD = 0.83) and 210 NH preschoolers (mean age = 4.64 years, SD = 0.83). All predictor and control variables were collected via teacher-reported questionnaires (self-compiled by the research team). Statistical analyses used hierarchical regression with separate models for each group. Results: For the CI group, after controlling for age and age of implantation, engagement in language learning activities emerged as the most stable and independent predictor of language ability. Auditory utilization status was another significant predictor, and its inclusion substantially increased the model’s R^2^. For the NH group, language ability was primarily predicted by age-related maturation, with language learning activities showing additional predictive value, while daily communication activities and learning attentional state contributed very little. Conclusions: Findings suggest that for educational rehabilitation of CI preschoolers: increasing engagement in language learning activities in classrooms may be more beneficial than relying on daily conversation or merely improving attention alone; meanwhile, auditory utilization skills should also be fostered synergistically. Importantly, language intervention models designed for NH preschoolers cannot be directly transferred to CI preschoolers.

## 1. Introduction

As newborn hearing screening and cochlear implantation have become increasingly accessible across China, the cohort of early implanted children has expanded significantly, and the average age of implantation continues to decline [1,2,3,4]. Advances in cochlear implant (CI) technology have enabled young children with hearing loss to receive effective auditory inputs during the sensitive window for language acquisition [5,6]. However, cochlear implantation is only the starting point for language development: young children’s language skills after surgery still need to be developed gradually through systematic training and support from the home and educational environments [7].

Some children who receive cochlear implants (CIs) at an early age can reach or even exceed the language proficiency levels of children with normal hearing (NH) in specific areas. For example, a study showed that congenitally deaf children who were fitted bilaterally in the 1st year of life can develop age-appropriate language skills by the time they start school [8], and CI children also achieved a level of skill in turn-taking, topic maintenance, and duration of topic maintenance which was similar to that of age- and language-matched NH children [9]. However, when compared to carefully matched NH peers, they often lag behind across multiple language domains, with documented areas of difficulty including speech perception in noise [10,11], productive grammatical morphology (e.g., bound morphemes and copulas) [12,13], acquisition of complex syntactic constructions [12], narrative microstructural skills (e.g., mean length of utterance and total number of words) [14], phonetic and phonological production skills [15], phonological processing ability [16,17] and vocabulary knowledge [18]. These findings have been documented across children aged 2 to 7 years, with the present study focused on specific evidence for the 3 to 6 year age range. However, language acquisition is associated with cognitive development [19], and early language input is a key factor facilitating children’s linguistic and cognitive development [20]. Research has demonstrated that language development during preschool years greatly influences later academic success and growth of social abilities [21]. Studies involving CI children also indicate that there exists a sensitive period for auditory rehabilitation, with an optimal window for implantation within the first 3.5–4.0 years of life (ideally before the second year), when central auditory pathways exhibit maximal plasticity to sound stimulation. The sensitive period eventually closes around 6.5–7.0 years of age, after which cortical reorganization becomes more limited. This neural normalization is strongly associated with key behavioral outcomes, typically resulting in superior auditory perceptual abilities such as distinguishing fine phonetic contrasts, understanding speech in noisy environments, and localizing sounds with greater accuracy [22]. Therefore, it is essential to identify and address modifiable factors that promote language development in children of this age group.

A report [23] notes that CIs have been validated in the medical field for their effects on auditory performance, and in the educational field for the positive impact of early implantation on language acquisition. However, the benefits of early implantation are not automatic, they depend critically on children’s active engagement in language interactions. Research has consistently shown that young children’s engagement in language activities is a core behavioral predictor of language development, with a study involving NH groups indicating that children primarily learn language through participation in purposeful turn-taking interactions [24]. Systematic reviews further confirm that engaging in rich dialog positively impacts children’s oral language, expressive vocabulary, and early reading skills in typically developing children [25]. NH group’s positive and negative engagement with teachers, peers, and tasks has been shown to uniquely predict early school success and vocabulary development [26,27,28]. Specifically, young children’s classroom engagement in language learning activities is directly correlated with their vocabulary skills [29]. Based on the evidence from NH group, it is reasonable to hypothesize that for children with hearing loss who use effective hearing aids (e.g., hearing aids or cochlear implants), increased participation in high-quality language activities may likewise enhance language development and could help bridge the language experience gap with their normal-hearing peers. However, this hypothesis requires direct empirical testing in the population of CI group.

Definitions of language activities vary across studies [30,31,32,33,34,35,36,37,38,39]. The definition used in this study was formulated by the authors based on a synthesis of the prior literature, semi-structured interviews, and the results of exploratory and confirmatory factor analyses. Accordingly, we define language activities as all interactions or processes centered on language use; in educational settings, these include teacher-structured language tasks and children’s spontaneous daily communications. In this study, “language activity” refers to a child’s behavioral engagement and initiative in these activities.

Current research indicates that in educational settings, in addition to structured rehabilitation training, children’s learning attentional states (e.g., attention span and level of engagement in language tasks) [40,41] and auditory functioning (e.g., the child’s perception of sound and ability to perceive and distinguish speech sounds) [42,43,44] are associated with language acquisition. These findings provide important insights into classroom-related factors. Although existing studies have separately examined the associations of learning attentional states, auditory functioning, and language activity participation with language ability, very few have included all these variables simultaneously in the same analysis, particularly by statistically controlling for the first two factors before testing the unique explanatory power of the third. For CI preschool children, this lack of net-effect analysis leaves an important question: can encouraging classroom interaction promote language development independently of the child’s own attitudes and auditory capacity? This study addresses this gap by simultaneously incorporating learning attentional states, auditory functioning, and the degree of participation in language activities, aiming to clarify the relative contributions of these factors to language ability.

Another underexplored question is whether the pattern of how these variables—learning attentional states, auditory functioning, and language activity participation—explain language ability differs fundamentally between CI preschoolers and their NH peers. Typically, language development in the latter follows a relatively stable trajectory, with age accounting for most variance [45]; however, for the former, their more limited auditory input may play a more critical role than age or SERS in shaping attentional states and fostering participation behaviors. By including an NH control group and performing hierarchical regression comparisons, we can distinguish which factors are universal predictors of language development, and which contribute uniquely to the hearing-impaired population. Such a comparison is essential for understanding the developmental pathways of language in CI preschool children and for designing differentiated educational interventions.

In summary, this study focused on CI preschool children in China, aiming to examine how actual language activities in educational settings relate to their language abilities. Accordingly, it quantitatively assesses the engagement of each language activity type along with other metrics of the educational language environment and then verifies the relationship between these language activity types and language ability. After controlling for age, age of implantation (CI group only), and socioeconomic status (SESR), we asked the following questions: How much additional variation in language ability can be explained by auditory utilization skills (CI group only), learning attentional state, and participation in language activities across two dimensions: participation in structured language learning activities and the extent of spontaneous daily communication? Does this pattern of explanatory power differ between the two groups? By contrasting these findings with those obtained from NH preschoolers, we seek to provide fundamental data for developing suitable and effective support strategies. To address these research questions, an observational scale of language learning activities suitable for CI and NH preschool children was self-developed in this study, in order to compare the characteristics of language activities in educational settings between the two groups. This instrument has passed validity and reliability testing; please refer to the Section 2 for the specific validation procedures.

## 2. Materials and Methods

### 2.1. Participants

The total sample for this study comprised 210 NH preschool children and 189 CI users, aged 3 years to 5 years and 11 months, with the latter split according to three implant types: bilateral (n = 118), unilateral (n = 31), and bimodal (n = 40). Of these, the 118 preschool children with bilateral implants and 135 NH preschool children were the same sample as that used in a previous study of home settings [46]. For the present study, children’s language ability was directly assessed, whereas data on language activities, learning attentional state, and auditory utilization status were collected via questionnaires completed by the teachers who instructed these children daily. For the CI group, a total of 74 teachers from 13 rehabilitation centers participated, with a mean age of 35.18 years (SD = 7.27), mean teaching experience of 10.25 years (SD = 6.06), mean experience working with hearing-impaired children of 8.21 years (SD = 5.37), and relevant teaching qualifications. For the NH group, a total of 16 preschool teachers participated, with a mean age of 28.25 years (SD = 4.73), mean teaching experience of 5.31 years (SD = 3.72), and kindergarten teaching qualifications. All participants were drawn from the collaborative research cohort of a project funded by the Japan Science and Technology Agency (JST) (Grant Number: JPMJSP2124). The CI group consisted of 114 males and 75 females, with a mean age of 4.53 years (standard deviation [SD] = 0.83 years) and a mean age at implantation of 1.98 years (range: 0.5 to 3.83 years). The NH group comprised 105 males and 105 females, with a mean age of 4.64 years (SD = 0.83 years). Participants’ main demographic characteristics are presented in Table 1.

The children in the two groups of this study attended two types of kindergartens: CI preschool children were recruited from one affiliated with a rehabilitation center, while NH preschool children attended regular public kindergartens. It should be noted that both types of kindergartens strictly followed the Kindergarten Education Guidelines (Trial Implementation) (Ministry of Education of China, 2001) [47] and organized all educational activities based on the five developmental domains specified in the guidelines, namely Health, Language, Society, Science, and Arts, which cover the core aspects of young children’s holistic development, and the related activity plans and developmental goals are essential components of early childhood education. According to the guidelines, the two types of kindergartens were highly comparable in terms of daily schedules, core activity formats (e.g., teacher-led whole-class instruction, free play, story-telling, music/rhythm activities, mealtime conversations, and outdoor activities), and time allocation for each activity. The systematic difference was that, in the CI kindergarten, each child typically received 1–2 additional sessions per day, which could be either individualized auditory–verbal therapy or small-group rehabilitative sessions depending on the daily schedule. All CI preschool children received standard auditory–verbal therapy (AVT) at their respective rehabilitation centers, including individual sessions and group activities, while the NH children received regular preschool education without specialized language therapy.

Participants in the CI group were systematically recruited from 13 rehabilitation centers across 12 locations, including Baoding City in Hebei Province; Chongqing Municipality; Guangzhou City in Guangdong Province; Huaihua City, Zhuzhou City, and Changsha City in Hunan Province; Nanchang City and Ganzhou City in Jiangxi Province; Zhengzhou City in Henan Province; Kunming City in Yunnan Province; Taizhou City in Zhejiang Province; and Beijing Municipality (the capital of China). Eligible children were aged 3 to 5 years 11 months, were users of CIs, and had at least one year of CI use, considering that auditory and language adaptation following implantation typically requires 6–12 months and that children’s auditory perception abilities generally stabilize approximately 12 months post-implantation [48]. Participants were also required to have no additional disabilities. To rule out general developmental delays, all children were assed using the Chinese version of the Ages & Stages Questionnaire, Third Edition (ASQ-3)—a standardized screening tool designed for children aged 1 to 5 years and 11 months that assesses five domains: communication, gross motor skills, fine motor skills, problem-solving, and personal–social development. Six items per domain are scored using a 10-point scale, yielding a maximum of 60 per domain and a total sum of 300. The questionnaires were completed by parents who relied on their observations of their children’s everyday conduct. To maintain sample consistency, the following selection criteria were applied: for the CI group, any child whose score fell substantially below the normative range in any ASQ-3 domain other than the communication domain (suggesting potential co-occurring developmental delays) was excluded. Ultimately, all 189 participants satisfied the inclusion criteria and were enrolled in the study. The Chinese version of the ASQ-3 has been validated in a normative Chinese population, showing strong psychometric properties with high reliability and validity, with a Cronbach’s alpha of 0.80 indicating good internal consistency and satisfactory item homogeneity [49]. Based on psychometric evidence, the Chinese version of the ASQ-3 was deemed appropriate for assessing developmental progress in the present study. A systematic recruitment process was used to obtain the NH group from a regular preschool in Jiangxi Province, China. Eligible participants were children aged 3 to 5 years 11 months whose hearing status was confirmed through parental reports and school health records, including annual physical examinations with professional audiological screening, with both sources indicating no known hearing impairments or other developmental disabilities. Children who scored substantially below the normative range in any ASQ-3 domain were excluded. However, all 210 NH preschool children met the inclusion criteria and were fully enrolled in the study. Ethical approval for this study was obtained from the Research Ethics Committee of the Faculty of Human Sciences, University of Tsukuba (Approval No. Tsukuba 2024-176A). Additionally, we obtained written informed consent from the school principal, teachers, and the parents of all participating children.

### 2.2. Assessment of Language Ability

The Mandarin Clinical Evaluation of Language for Preschoolers’ Core Scale (MCELP-CS) [50] was administered to assess participants’ language abilities in this study and was selected based on several considerations: its established reliability and validity, as confirmed by prior research [51] (Cronbach’s α > 0.70, ICC > 0.75); its strong convergent validity, evidenced by significant correlations with the PPVT-R; its grounding in the cultural and educational context of Chinese preschoolers; and its demonstrated diagnostic accuracy in identifying language disorders among children with autism, cerebral palsy, and hearing impairment.

The MCELP-CS assesses receptive and expressive language in children aged 3 to 5 years 11 months and comprises five subtests: vocabulary comprehension, sentence comprehension, vocabulary naming, sentence structure imitation, and story retelling. Individual assessments are conducted in rehabilitation centers or nursery classrooms. Before the assessment, the examiner provides a brief, easy-to-understand verbal explanation lasting approximately five minutes and sets out the tasks to be performed. Taking into account the children’s attention span and stamina, and to ensure the quality of the assessment whilst minimizing the effects of fatigue, the five subtests of the MCELP-CS are divided into three separate testing sessions, which all children complete in a fixed, standardized order: Session 1: vocabulary comprehension (10–20 min); Session 2: sentence comprehension and vocabulary naming (15–20 min); Session 3: sentence structure imitation and story retelling (15–20 min). The total assessment time spanned 40 to 60 min, divided over different days.

### 2.3. Basic Information and Language Activities Questionnaire in the Educational Setting

The questionnaire was designed based on the domestic and international literature concerning the linguistic environments of educational settings for NH and CI groups. To enhance the questionnaire’s applicability within the Chinese cultural context and educational settings for CI users, the researchers first conducted preliminary interviews (lasting approximately 15–20 min each) with teachers of both the CI and NH group (30 from each group) to understand the types of language activities that occur in educational settings. Based on the literature review and the interview findings, we developed a preliminary version of the questionnaire comprising three main parts: demographic information, a measure of learning attentional states and auditory utilization status, and a self-developed eight-item scale measuring language activities in educational settings. The second part draws on a questionnaire used in a study by a Japanese expert in hearing education [23], while the third part is the self-developed scale for language activities in educational settings.

The development of items for language activities in educational settings was conducted by integrating two sources: Chinese educational policy documents and qualitative data from preliminary surveys. First, we referred to the “Guidelines for Kindergarten Education (Trial)” [47] and the “Guidance on Learning and Development for Children Aged 3–6” [52], which serve as guidelines for early childhood education in China. These documents present five major forms of educational activities in the language domain: “conversation activities,” “narrative activities,” “listening and speaking game activities,” “literature study activities,” and “early reading activities.” The scale used in this study faithfully reflects this official educational framework and includes corresponding items. Next, to determine the specific content and evaluation focus of these items, we utilized the results of interviews with kindergarten teachers conducted during a preliminary survey. Through this process, the scale items were grounded not only in ideal educational plans but also in actual classroom practices and children’s behaviors. Furthermore, in language development, particularly regarding the communication abilities of CI preschoolers, everyday social interaction is extremely important, not just formal participation in learning activities. Therefore, in addition to the five structured activities mentioned above, we added three items: interaction among children, interaction between children and teachers/staff, and clarity of self-expression. Through this, we aimed to comprehensively evaluate the language activities in educational settings from two perspectives: planned learning activities and spontaneous social interactions. The initial questionnaire’s content validity was established through expert evaluation. Based on their input, items that were repetitive or unclear were eliminated, resulting in the retention of the 8 most representative language activity items. Before launching the formal survey, we pilot-tested the questionnaire on six teachers to ensure question clarity, lack of ambiguity, and ease of comprehension.

To explore the underlying factor structure of the original items and to reduce the complexity of the measurement tool, an exploratory factor analysis (EFA) was conducted on 135 valid questionnaires that were randomly collected from teachers of Chinese kindergarten children between the ages of 3 and 5 years 11 months. Both the KMO (0.82) and Bartlett’s sphericity test (χ^2^ = 413.032, df = 28, *p* < 0.001) confirmed that the data met the requirements for factor analysis.

After data analysis, we refined the items to further streamline the model and enhance the scale’s validity. Two distinct factors were ultimately extracted via principal component analysis (varimax rotation), guided by eigenvalue criteria, scree plot inspection, and factor interpretability, leading to the retention of eight items. Factor 1 comprises five items, including conversation activities (e.g., morning talk, sharing after play, and theme-based discussions), narrative activities (e.g., telling picture book stories and describing pictures), listening–speaking games (e.g., word games and riddles), literary learning activities (e.g., nursery rhymes, ancient poems, and prose), and book reading activities (e.g., picture book reading in the book corner), named “language learning activities in educational settings.” Factor 2 comprises three items, primarily covering the child’s degree of interaction with other children and with teachers or other staff in the kindergarten, and the extent to which the child can clearly express his/her own needs using language, named “Daily communication activities in educational settings.” Together, these two factors explained 62.59% of the total variance.

After that, we tested this two-factor model with a new sample of 210 valid questionnaires, using confirmatory factor analysis (CFA). The model demonstrated an acceptable fit to the data: χ^2^ (19) = 51.828, χ^2^/df = 2.728, CFI = 0.944, TLI = 0.912, RMSEA = 0.091 (90% CI [0.062, 0.121]), SRMR = 0.049, and NNFI = 0.912. All standardized factor loadings were statistically significant, ranging from 0.478 to 0.841. Regarding discriminant validity, the Pearson correlation coefficient between the two factors was 0.555, which is smaller than the square roots of their respective AVEs (0.695 and 0.632), indicating good discriminant validity. Regarding reliability, Cronbach’s α values were 0.82 and 0.65, and composite reliability (CR) was 0.82 and 0.67, respectively. The second factor consists of only three items, which means it tended to yield slightly lower reliability estimates; nevertheless, for exploratory research, α = 0.65 and CR = 0.67 are considered acceptable (above the commonly used threshold of 0.60).

Regarding validity, both construct validity (supported by the good model fit) and discriminant validity (as shown above) were established. For convergent validity, although the average variance extracted (AVE) for the two factors was 0.400 and 0.482, respectively, falling below the benchmark value of 0.50, from a methodological perspective, the CR values exceeded the acceptable minimum of 0.60, and all factor loadings were significant and of moderate to high magnitude (0.48–0.84). Thus, convergent validity in the exploratory phase of this measurement model can be deemed sufficient due to the fact that the CR is relatively insensitive to measurement error, indicating that the indicators consistently reflect the underlying construct [53,54,55].

Thus, the final questionnaire consists of three parts. The first part collects background information from participants, such as age, age of cochlear implantation, gender, presence of additional disabilities, CI usage status, hearing levels before and after implantation, family economic level, parents’ educational levels (father and mother separately), and the number of books at home (including those owned by the child and adults). To streamline the model and capture overall family resources, we created composite variables by combining related items. Specifically, the educational levels of the father and mother were combined into a single “parental education level” variable (calculated as the average; Cronbach’s α = 0.81, CITC > 0.68), and the number of children’s books and adult books at home was averaged to form a composite “number of books in the home” variable (Cronbach’s α = 0.67, CITC > 0.5). Both composite measures were obtained from 5-point Likert scale items and subsequently used in the analyses.

The second part assesses two dimensions: auditory utilization status and learning attentional state. The former comprised three items: (1) awareness of sounds, (2) perception of speech sounds, and (3) the child’s ability to listen attentively to teachers and peers. The latter also comprised three items: (1) the degree of distractibility, (2) the duration of sustained attention, and (3) the extent to which the child shows confusion or hesitation when following oral instructions. Each item was rated on a 5-point Likert scale (1 = never to 5 = always), with higher scores indicating better auditory utilization and learning attentional state. Auditory utilization status differs from learning attentional state and communication ability: learning attentional state refers to general cognitive processes (e.g., distractibility and sustained attention) that are not limited to auditory input, whereas auditory utilization specifically involves active monitoring and use of sound information (e.g., speech perception and attentive listening) [23].

The third part measures variables related to the level of language activities within educational settings and consists of a two-dimensional scale (including the eight items previously mentioned: language learning activities in educational settings and daily communication activities in educational settings), using a 5-point Likert scale for scoring. The full questionnaire is available in Supplementary File S1 for reference.

### 2.4. Data Analysis

The primary objective of this study was to investigate the association between language activities within educational settings and the language abilities of CI preschool children, as well as to compare these findings with those of a control group consisting of NH preschoolers. All statistical procedures were performed using IBM SPSS Statistics, Version 30.0. For each variable, descriptive statistics were calculated, with continuous variables expressed as means ± standard deviations. To assess the normality of the data, both the Shapiro–Wilk test and Q-Q plots were applied, under the assumption of a normal distribution, which was confirmed for each age subgroup as well as for the full datasets of both groups. Consequently, group comparisons were carried out using independent-samples *t*-tests (or Welch’s *t*-test when the assumption of homogeneity of variances was violated). To adjust for multiple comparisons across the three age strata, all *p*-values were corrected using the Bonferroni method. Pearson’s correlation coefficient was used to evaluate the relationships between variables in both groups.

To further explore the associations between language activities, other family-related variables, and language ability, we conducted separate stratified regression analyses for the CI and NH groups. Initial analyses showed moderate to high correlations among parental education level, family income, and the number of books at home (Pearson’s r ranging from 0.421 to 0.545), with variance inflation factors (VIFs) for the regression model variables between 1.37 and 1.67. To improve model stability, reduce the potential impact of multicollinearity on parameter estimates, and align with the theoretical view that these variables collectively reflect family socioeconomic status, we constructed a composite variable labeled SESR. This composite was derived from three ordinal indicators—the highest parental education level, annual household income bracket, and the number of books at home—and created by applying principal component analysis (PCA) rather than using a simple average, which would assume equal spacing between ordinal categories. The suitability of the data for dimensionality reduction was confirmed by a Kaiser–Meyer–Olkin (KMO) value of 0.67 and a significant Bartlett’s test of sphericity (*p* < 0.001). A single component with an eigenvalue exceeding 1 (eigenvalue = 1.934) was retained, explaining 64.47% of the total variance, and all three indicators loaded strongly onto this component (parental education level: 0.829; family income: 0.754; number of books: 0.824). The component scores derived from this first principal component were saved and employed as a continuous SESR variable in all subsequent analyses. This data-driven, weighted composite captures the shared variance among the indicators while avoiding the assumption of equal intervals inherent in the original ordinal scales. For the CI group, after adjusting for age, SESR, age of cochlear implantation, auditory utilization status, and learning attentional state, we assessed the effects of language learning activities and daily communication activities in educational settings on language ability (including daily communication activities first, followed by language learning activities). For the NH group, the effects of the same activities on language ability were assessed after controlling for age, SESR, and learning attentional state. In addition, to examine the effect of the order in which language activity variables were included on the results of the hierarchical regression, we repeated the analysis after swapping the order of the predictor variables (including language learning activities first, followed by daily communication activities). A significance threshold of *p* < 0.05 was adopted for all regression models and predictor variables. The change in R^2^ was used to evaluate the increase in the model’s explanatory power resulting from the addition of predictor variables, and the relative contribution of each predictor was indicated by its unstandardized coefficient (β). Detailed results of the analyses are shown in the table below.

## 3. Results

### 3.1. Language Ability, Language Activity, and Other Variables in Educational Settings

Table 2 presents the descriptive statistics and independent-samples *t*-test results comparing the CI and NH groups across various variables, with data stratified by age (3, 4, and 5 years).

For language abilities, the NH group demonstrated significantly better performance than the CI group across all three age levels, with large effect sizes (3-year-olds: t (90.55) = 5.65, *p* < 0.001, d = 1.02; 4-year-olds: t (95.73) = 10.66, *p* < 0.001, d = 1.88; 5-year-olds: t (68.38) = 7.04, *p* < 0.001, d = 1.30). Correspondingly, the mean auditory utilization status also increased across age groups, from 11.87 (SD = 2.62) at age 3 to 12.27 (SD = 2.59) at age 5. There were no significant differences between the CI and NH groups in SESR and learning attentional state at any age after Bonferroni adjustment (all *p* > 0.05). In language learning activities within educational settings, CI group scored lower than those in the NH group across all age groups (age 3: t (90.03) = −2.32, *p* = 0.018, d = 0.42; age 4: t (113.92) = −1.84, *p* = 0.07, d = 0.32; age 5: t (104.96) = −1.54, *p* = 0.12, d = 0.27). For daily communication activities in educational settings, the CI group also scored significantly lower than the NH group at all ages (age 3: t (91.14) = −2.33, *p* = 0.017, d = 0.42; age 4: t (111.16) = −2.89, *p* = 0.004, d = 0.50; age 5: t (82.05) = −2.35, *p* = 0.016, d = 0.43). Overall, the results show that children in the CI group show lower performance than those in the NH group in both language learning and daily communication activities in educational settings, with statistically significant differences primarily at younger ages for engagement in language learning activities and across all examined ages for communication activities.

### 3.2. The Correlation Between Language Ability and Variables in Educational Settings

Table 3 and Table 4 present the results of the correlation analysis between language ability and other variables in educational settings for the CI group and NH group, respectively. Language abilities were significantly positively correlated with age (r = 0.355, *p* < 0.01), SESR (r = 0.268, *p* < 0.01), auditory utilization status (r = 0.631, *p* < 0.01), learning attentional state (r = 0.516, *p* < 0.01), language learning activities in educational settings (r = 0.683, *p* < 0.01), and daily communication activities in educational settings (r = 0.642, *p* < 0.01). In contrast, they were significantly negatively correlated with age of cochlear implantation (CI age: r = −0.211, *p* < 0.01). Furthermore, auditory utilization status, learning attentional state, language learning activities, and daily communication activities were all significantly intercorrelated, with coefficients ranging from r = 0.500 to r = 0.745 (all *p* < 0.01), indicating moderate to strong positive associations among these variables. Compared to the CI group, the NH group exhibited distinct patterns of correlations between language abilities. Language abilities in the NH group were significantly positively correlated with age (r = 0.735, *p* < 0.01), learning attentional state (r = 0.387, *p* < 0.01), language learning activities in educational settings (r = 0.378, *p* < 0.01), and daily communication activities in educational settings (r = 0.377, *p* < 0.01). However, they were not significantly correlated with the composite index of SESR (r = 0.116, *p* > 0.05). Learning attentional state, language learning activities, and daily communication activities were all significantly intercorrelated (all *p* < 0.001), indicating moderate to strong positive associations among these variables in the NH group.

### 3.3. The Impact of All Variables in the Educational Settings on Language Ability

To control for the influence of age, CI age of implantation, and other relevant factors, we then conducted a hierarchical multiple linear regression analysis in the CI and NH groups (see Table 5 and Table 6). In the CI group, six models were estimated, with predictors entered sequentially. Model 1 included two predictors, age and CI age of implantation, and was significant (F = 26.658, *p* < 0.01), explaining 22.3% of the variance (R^2^ = 0.223, adjusted R^2^ = 0.214). Age (*β* = 29.43, SE = 4.51, *p* < 0.01) and CI age of implantation (*β* = −21.07, SE = 4.38, *p* < 0.01) were both significant predictors, with a younger implantation age associated with better outcomes. Model 2 added SESR and remained significant (F = 22.676, *p* < 0.01), explaining 26.9% of the variance (R^2^ = 0.269, adjusted R^2^ = 0.257). The change in R^2^ was significant (Delta R^2^ = 0.046). Age (*β* = 29.05, *p* < 0.01), CI age of implantation (*β* = −17.63, *p* < 0.01), and SESR (*β* = 2.57, *p* = 0.001) were all significant predictors. Model 3 introduced auditory utilization status and accounted for 54.1% of the variance (R^2^ = 0.541, adjusted R^2^ = 0.531), with a significant change in R^2^ of 0.272 (F = 54.177, *p* < 0.01). All four predictors were significant: age (*β* = 24.41, *p* < 0.01), CI age of implantation (*β* = −9.35, *p* = 0.009), SESR (*β* = 1.60, *p* = 0.009), and auditory utilization status (*β* = 11.37, *p* < 0.01). Model 4 added learning attentional state and was significant (F = 45.558, *p* < 0.01), explaining 55.5% of the variance (R^2^ = 0.555, adjusted R2 = 0.542). The R^2^ change of 0.014 was small but significant (*p* = 0.019). Age (*β* = 22.86, *p* < 0.01), CI age of implantation (*β* = −9.11, *p* = 0.010), SESR (*β* = 1.46, *p* = 0.016), auditory utilization status (*β* = 9.75, *p* < 0.01), and learning attentional state (*β* = 3.57, *p* = 0.019) were all significant. Model 5 included daily communication activities in educational settings and explained 60.2% of the variance (R^2^ = 0.602, adjusted R^2^ = 0.589), and the R^2^ change was 0.047 (F = 45.868, *p* < 0.01). Age (*β* = 21.06, *p* < 0.01), CI age of implantation (*β* = −7.99, *p* = 0.018), SESR (*β* = 1.23, *p* = 0.034), auditory utilization status (*β* = 6.34, *p* < 0.01), and daily communication activities (*β* = 5.61, *p* < 0.01) were significant; however, learning attentional state was no longer significant (*β* = 2.43, *p* = 0.094). Model 6 added language learning activities in educational settings as the final predictor; this full model was significant (F = 47.530, *p* < 0.01) and accounted for 64.8% of the variance (R^2^ = 0.648, adjusted R^2^ = 0.634). The addition of language learning activities resulted in a significant increase in R^2^ of 0.046 (*p* < 0.01), indicating that this variable contributed unique variance beyond the previous predictors. In the final model, significant predictors were age (*β* = 20.60, *p* < 0.01), CI age of implantation (*β* = −9.01, *p* = 0.005), SESR (*β* = 1.16, *p* = 0.033), auditory utilization status (*β* = 5.18, *p* < 0.01), and language learning activities in educational settings (*β* = 3.85, *p* < 0.01), whereas learning attentional state (*β* = 0.94, *p* = 0.504) and daily communication activities (*β* = 2.11, *p* = 0.119) were not significant. All variance inflation factor (VIF) values were below 2.7, indicating no serious multicollinearity.

To examine the effect of variable (language activity) entry order on the hierarchical regression results, we reversed the order of predictors (entering language learning activities first, followed by daily communication activities) and compared the results with the original order (daily communication activities first, then language learning activities). With both orders, the full model (including both predictors) explained a total of ΔR^2^ = 0.093, and the overall model was significant (F(7181) = 47.530, *p* < 0.001).

Original order (daily communication first, then language learning): In Model 5, daily communication activities accounted for ΔR^2^ = 0.047, ΔF(1182) = 21.680, and *p* < 0.001; in Model 6, after adding language learning activities, ΔR^2^ = 0.046, ΔF(1181) = 23.490, and *p* < 0.001. In the final full model, the standardized coefficient for daily communication activities was not significant (β = 2.11, *p* = 0.119), whereas that for language learning activities was significant (β = 3.85, *p* < 0.001).

Reversed order (language learning first, then daily communication): In Model 5, language learning activities alone explained ΔR^2^ = 0.088, ΔF(1182) = 45.034, and *p* < 0.001; in Model 6, after adding daily communication activities, ΔR^2^ = 0.005, ΔF(1181) = 2.455, and *p* = 0.119, which was not significant. In the final full model (identical to that in the original order), daily communication activities remained non-significant (β = 2.11, *p* = 0.119), while language learning activities remained significant (β = 3.85, *p* < 0.001).

Taken together, language learning activities provided a significant increment in R2 after controlling for daily communication activities (ΔR^2^ = 0.047, *p* < 0.001), whereas daily communication activities did not provide a significant increment after controlling for language learning activities (ΔR^2^ = 0.005, *p* = 0.119). Moreover, regardless of entry order, only language learning activities showed a significant unique contribution in the full model. Thus, the apparent association between daily communication activities and language ability can be fully accounted for by their shared variance with language learning activities, which have a robust and unique independent contribution.

In the NH group, five models were tested. Model 1 only included age, which significantly predicted language ability (β = 0.74, *p* < 0.01), and explained 54.1% of the variance (R^2^ = 0.541, adjusted R^2^ = 0.538, F = 244.75, *p* < 0.01). Model 2 added the SESR; age remained significant (β = 0.73, *p* < 0.01), and the SESR also showed a significant positive effect (β = 0.09, *p* = 0.047). The change in R^2^ was 0.009, overall model F = 126.12, and *p* < 0.01, with total R^2^ = 0.549. Model 3 introduced learning attentional state; age (β = 0.68, *p* < 0.01) and learning attentional state (β = 0.22, *p* < 0.01) were significant, but the SESR became non-significant (β = 0.05, *p* = 0.241). The model accounted for 59.2% of the variance (R^2^ = 0.592, adjusted R^2^ = 0.586), ΔR^2^ = 0.043, F = 99.58, and *p* < 0.01. Model 4 added daily communication activities in educational settings; age (β = 0.66, *p* < 0.01), learning attentional state (β = 0.16, *p* = 0.001), and daily communication (β = 0.13, *p* = 0.009) were significant predictors, while the SESR remained non-significant (β = 0.05, *p* = 0.278) and R^2^ increased to 0.605 (adjusted R^2^ = 0.598, ΔR^2^ = 0.014, F = 78.62, *p* < 0.01). Model 5 further included language learning activities in educational settings; age (β = 0.66, *p* < 0.01) and language learning activities (β = 0.17, *p* = 0.002) were significant, while learning attentional state became marginally significant (β = 0.12, *p* = 0.023) and composite index (β = 0.03, *p* = 0.476) and daily communication (β = 0.06, *p* = 0.273) were not significant. The final model explained 62.4% of the variance (R^2^ = 0.624, adjusted R^2^ = 0.614, ΔR^2^ = 0.018, F = 67.57, *p* < 0.01). Overall, age consistently emerged as the strongest predictor across all models. Learning attentional state and language learning activities in educational settings contributed unique variance, whereas the SESR and daily communication activities became non-significant after controlling for other variables.

To examine the effect of variable (language activity) entry order on the hierarchical regression results, we reversed the order of predictors (entering language learning activities first, followed by daily communication activities) and compared the results with the original order (daily communication activities first, then language learning activities). In both orders, the full model (including both predictors) explained a total of ΔR^2^ = 0.032, and the overall model was significant (F(5204) = 67.568, *p* < 0.001).

Original order (daily communication first, then language learning): In Model 4, daily communication activities accounted for ΔR^2^ = 0.014, ΔF(1205) = 7.017, and *p* = 0.009; in Model 5, after adding language learning activities, ΔR^2^ = 0.018, ΔF(1204) = 9.830, and *p* = 0.002. In the final full model, the standardized coefficient for daily communication activities was not significant (β = 1.20, *p* = 0.273), whereas that for language learning activities was significant (β = 1.85, *p* = 0.002).

Reversed order (language learning first, then daily communication): In Model 4, language learning activities alone explained ΔR^2^ = 0.029, ΔF(1205) = 15.927, and *p* < 0.001; in Model 5, after adding daily communication activities, ΔR^2^ = 0.002, ΔF(1204) = 1.206, and *p* = 0.273, which was not significant. In the final full model (identical to that in the original order), daily communication activities remained non-significant (β = 1.20, *p* = 0.273), while language learning activities remained significant (β = 1.85, *p* = 0.002).

Taken together, language learning activities provided a significant increment in R^2^ after controlling for daily communication activities (ΔR^2^ = 0.018, *p* = 0.002), whereas daily communication activities did not after controlling for language learning activities (ΔR^2^ = 0.002, *p* = 0.273). Moreover, regardless of entry order, only language learning activities showed a significant unique contribution in the full model. Thus, the apparent association between daily communication activities and language ability can be fully accounted for by their shared variance with language learning activities, which have a robust and unique independent contribution.

## 4. Discussion

In the correlation and hierarchical regression analyses conducted separately for the CI and NH groups, we found that although the two groups shared some common factors associated with language ability, they showed notable differences in the association patterns of key variables. Specifically, the relationships between language ability and individual background factors, family resources (SESR), auditory utilization status, and educational environment variables were not identical across the two groups. Due to the cross-sectional design of this study, the reported relationships should be interpreted as correlational rather than causal, and no direction of causality can be inferred. These results suggest that language development under different hearing conditions follows both shared patterns of association and distinct correlational structures—findings that will be addressed in the following discussion.

The study showed that children in the CI group had significantly lower overall language abilities than those in the NH group at all three age points (3, 4, and 5 years), with all effect sizes classified as large. This reflects a clinically meaningful gap between CI and NH preschool children, and whereas the small to medium effect sizes for other variables suggest that group differences are minimal in this sample. This finding is consistent with previous research, indicating that even with CI placement, children may still lag behind their hearing peers in language comprehension and expression [12,13,14,15,16,17,18]. Regarding family background variables, there were no significant differences between the two groups in the SESR, indicating that the samples are well comparable with respect to core family resources.

The correlation analyses showed that in the CI group, language ability was positively associated with auditory utilization, learning attentional state, and language-related educational activities, but negatively associated with age of implantation. In contrast, NH children exhibited weaker or non-significant correlations with these variables and no association with the SESR. These findings suggest that the factors associated with language development may differ between the two groups, with the CI group showing stronger correlations with structured language input and active engagement. However, correlation alone does not imply causation; the hierarchical regression results below further clarify the unique predictive power of these variables.

Hierarchical regression results show that across both groups, age was consistently the strongest and most stable predictor, which is consistent with prior research [46]. This was particularly evident in the NH group, where age alone accounted for 54.1% of the variance in language ability (Model 1) and remained highly significant in all subsequent models. This pattern is consistent with maturational theories of language development, suggesting that as children grow older, they tend to accumulate experience and skills in areas such as vocabulary, syntax, and pragmatics. In the CI group, in addition to age, age of implantation showed a significant negative predictive association across all models, indicating that earlier implantation was related to better language ability—a finding consistent with the “sensitive period hypothesis for early auditory experience” [22] and earlier implantation optimum language development [56,57], highlighting the important role of auditory input during critical developmental windows for establishing central auditory pathways and language systems. Notably, after controlling for auditory utilization status and educational environment variables, although the predictive strength of implantation age decreased, it remained significant, suggesting that its association with language ability is independent and not fully accounted for by subsequent variables.

Unlike previous studies that reported a strong direct association between the SESR and language outcomes [58], in the CI group, the SESR was significant in Models 2 through 4; however, although it remained significant in the final model, its contribution to the explained variance was relatively small. In the NH group, the SESR was only marginally significant in Model 2 and became non-significant after learning attentional state was included, suggesting that the direct statistical contribution of family resources on language proficiency is relatively limited. One possible interpretation is that the association of the SESR with language ability may be more indirectly reflected; for example, it could be statistically related to or accounted for by a rich language input environment, educational methods, and learning support, and these pathways may be partially accounted for by variables such as “auditory utilization,” “learning attentional state,” and “language learning activities” in this study.

In the CI group, the inclusion of auditory utilization status in Model 3 was associated with the largest increase in R^2^, and this variable remained significant in all subsequent models, including the final one. This finding has important clinical and educational implications: even with a cochlear implant, the ability to consistently and actively use the auditory pathway for language reception and communication in daily life is a key behavioral factor that is strongly associated with individual differences in language ability. In contrast, because the NH group possesses a complete auditory pathway from birth, this variable was not included in the analysis, further underscoring the strong association between the CI group’s language ability and their acquired auditory behavior. Learning attentional state was significant in Model 4 but became non-significant after “daily communication activities” were added, and remained so in the final model. In the NH group, by contrast, learning attentional state was significant in both Models 3 and 4, and remained marginally significant even in the final model. This difference suggests that, among the CI group, the association between learning attentional state and language ability may be accounted for by more concrete behavioral variables (such as actual participation in communication and language learning activities), whereas among the NH group, learning attentional state, as a relatively stable personal trait, shows a unique association with language ability that is not fully explained by these behavioral measures.

Moreover, in the CI group, “daily communication activities” were significant in Model 5 but became non-significant after “language learning activities” were included; meanwhile, the latter were significant in the final model. A similar pattern emerged in the NH group: “daily communication” was significant in Model 4 but became non-significant after being replaced by “language learning activities,” and the latter became the only significant educational environment variable in the final model. These findings offer important pedagogical implications: in educational settings, structured language learning activities show greater incremental validity in their association with children’s language abilities than everyday oral communication. One possible interpretation is that language learning activities tend to involve more complex syntactic structures and a richer vocabulary, all of which are associated with language development in ways that differ from natural conversation.

Based on the above, language development in the CI group is associated with multiple factors, including biological factors (age and age of implantation), family resources, auditory behavior, and educational environment, with auditory utilization status showing the strongest unique association. In the NH group, language development is most strongly associated with age, while the additional explanatory power of other variables is relatively small, suggesting that age-related factors are the primary correlates in the typical developmental trajectory. Language learning activities emerged as a predictor for both groups, indicating that systematic language learning experiences in educational settings are closely associated with language ability. The association of daily communication activities with language ability was no longer significant after controlling for language learning activities, suggesting that its apparent relationship may be accounted for by language learning activities rather than representing a unique independent contribution.

## 5. Research Limitations

When interpreting these findings, we must clearly acknowledge certain limitations. This study utilized cross-sectional data, with all variables measured at a single point in time, making it impossible to determine the direction of causality between predictive factors and language proficiency. For example, language learning activities and language development may be positively associated; it is possible that such activities support development, or that children with stronger skills are more likely to engage in them. Future research should employ longitudinal designs or cross-lagged analyses to examine causal relationships. Second, all independent variables (language activities, use of hearing, and learning attentional states) were measured using questionnaires completed by teachers. While teachers are knowledgeable informants, relying on a single reporter for multiple constructs may introduce common method variance (CMV) [59], which can artificially inflate correlations; thus, our reported associations might be overestimated. We did not perform a statistical test for CMV (e.g., Harman’s single-factor test), which is a limitation. Future research should include objective measures such as direct classroom observations (e.g., structured coding of language activities and attentional states), video coding, or LENA (Language Environment Analysis) devices to reduce method-induced inflation, which would help decouple the measurement of predictors and outcomes in future longitudinal or experimental designs. Third, although this study controlled several key factors, unmeasured variables (such as the quality of rehabilitation, cochlear implant characteristics, and tuning parameters) may be associated with the results. Future studies could adopt a longitudinal design to track long-term effects, incorporate objective measurement tools (such as recordings of language environments) to reduce reporting bias, and further explore the specific cognitive mechanisms through which language activities in educational settings influence language proficiency. Although the direction of causality remains to be determined, the strong association between language learning activities, auditory processing skills, and language ability provides valuable insights for school education and early intervention. Fourth, this study is based on the method used to assess hearing status in the NH control group. Although we combined teacher reports with the results of professional hearing screenings conducted during the school’s annual physical examinations, this approach remains essentially a screening tool rather than a comprehensive audiological diagnosis. Since standardized pure-tone audiometry was not performed in a controlled acoustic environment, we cannot completely rule out undetected mild or unilateral hearing loss in the control group. In future studies, where conditions permit, standardized audiological assessments should be conducted for all participants (including the control group) to more rigorously ensure accurate group classification and enhance the reliability of the conclusions. Fifth, a limitation of this study is the moderate to high correlations among language learning activities, daily communication, auditory utilization, and attention. Although high shared variance may raise concerns about conceptual overlap, we argue that it largely reflects theoretically expected relationships (e.g., attention → auditory utilization → language learning) and common method bias. Discriminant validity tests confirmed that the most confusable pair, language learning activities and daily communication, are empirically distinguishable (HTMT = 0.749 < 0.85). Auditory utilization and attention are widely recognized as distinct constructs in the literature and thus did not require additional testing. Future research should employ multi-source data or experimental designs to reduce common method bias. Finally, the CI and NH groups were taught by different teacher teams from different settings, with no shared teachers. Teacher age and experience differed between groups but were confounded with group assignment, precluding statistical control. Sex distribution was slightly unbalanced (more boys in the CI group), and some of the literature suggests slower language development in boys, making sex a potential confounder. For children with bilateral CIs, we did not distinguish simultaneous versus sequential implantation. Additionally, some children had language ages below 3 years; we retained them to avoid selection bias (typical early post-implant lag), but this increased statistical error.

## 6. Conclusions

In summary, this study reveals distinct predictive patterns of educational language activities in relation to language ability for preschool CI and NH groups. The CI group lag behind the NH group in overall language ability, but earlier implantation is associated with stronger language skills, which highlights the importance of early intervention. Although the CI group showed no difference in participation in language learning activities by age 5, gaps in language abilities still remained. After controlling for age, age of implantation, SESR, auditory utilization, and learning attentional state, language activities in educational settings show a significant and independent predictive association with language outcomes in this group. Notably, auditory utilization status, defined as children’s proactive use of the auditory channel for learning and communication in daily educational settings, emerges as a key predictor, and its inclusion substantially increases model explanatory power.

For the CI group, both daily communication activities and language learning activities in educational settings are significantly associated with language ability. However, after controlling for auditory utilization and other variables, language learning activities (e.g., shared reading, word games, and story retelling) show the most robust and independent predictive effect, whereas the predictive roles of daily communication and learning attentional state become non-significant. This suggests that in educational settings, structured language learning activities are more strongly predictive of their language development than free conversation or general learning attentional states. Importantly, although auditory utilization makes a significant contribution, its predictive effect may be partially shared with or supplemented by language learning activities; the two are not substitutes but may jointly predict language development. In sharp contrast, the pattern for the NH group is markedly different. Language learning activities are the significant educational language activity variable, while daily communication and learning attentional state contribute little. The group’s language ability is primarily predicted by age-related maturation, with language learning activities providing additional predictive value.

This study offers three insights for CI preschool children’s rehabilitation: (1) Given the strong associations, it may be beneficial to prioritize increasing children’s engagement in structured language learning activities (e.g., shared reading, word awareness games, and narrative exercises) rather than relying mainly on daily free conversation or merely improving learning attention. (2) Auditory utilization status should be a key variable to consider in intervention design. (3) Language intervention models designed for NH groups are not directly transferable to CI groups. The synergistic associations of enhancing engagement in language learning activities while fostering auditory utilization skills deserve attention in practice.

## Figures and Tables

**Table 1:** Participants’ information.

		CI Preschool Children (n = 189)	NH Preschool Children (n = 210)
Age	3	62	70
	4	65	70
	5	62	70
Gender	Boys	114 (60.32%)	105 (50%)
	Girls	75 (39.68%)	105 (50%)
Average Age		4.53 (0.83)	4.64 (0.83)
CI Age of Implantation		1.98 (0.84)	
CI Status (Bilateral)		118 (62.43%)	-
CI Status (Bimodal)		40 (21.16%)	
CI Status (Unilateral)		31 (16.40%)	

Note: CI: cochlear implant; NH: normal-heating. Age range for CI placement (minimum age 0.5 years and maximum age 3.83 years).

**Table 2:** Age-stratified descriptive data and independent *t*-test results examining group differences in variables. (Format adapted from He & Chung, 2026 [46]).

		CI Group (n = 189)		NH Group (n = 210)						
	Age	Mean (SD)	N	Mean (SD)	N	Levene’s Test	*t*	df	*p*-Value/Adjusted *p*-Value	Effect Size
Language abilities	3	87.96 (50.23)	62	128.31 (26.80)	70	**33.59**	5.65	90.55	0.000 **	1.02
	4	101.57 (45.27)	65	168.79 (24.01)	70	**25.66**	10.66	95.73	0.000 **	1.88
	5	135.85 (57.78)	62	189.07 (15.09)	70	**69.96**	7.04	68.38	0.000 **	1.30
Age of implantation	3	1.66 (0.65)	62	−	−	−	−	−	−	−
	4	2.10 (0.82)	65							
	5	2.18 (0.93)	62							
Composite index (SESR)	3	12.42 (4.68)	62	9.33 (3.35)	70	3.14	0.26	130.00	0.80	0.05
	4	12.57 (4.41)	65	12.10 (3.49)	70	**5.20**	0.68	121.80	0.49	0.12
	3	12.02 (5.06)	62	12.53 (4.43)	70	1.84	-0.62	130.00	0.54	0.11
Auditory utilization status	3	11.87 (2.62)	62	−	−	−	−	−	−	−
	4	11.95 (2.72)	65							
	5	12.27 (2.59)	62							
Learning attentional state	3	9.31 (1.83)	62	9.41 (2.05)	70	1.41	−0.317	130.00	0.75	0.06
	4	9.65 (2.27)	65	10.14 (2.32)	70	0.34	−1.257	133.00	0.21	0.22
	5	10.31 (2.47)	62	10.64 (1.89)	70	**4.09**	−0.871	113.69	0.38	0.15
Language learning activities in educational settings	3	15.00 (4.86)	62	16.60 (2.57)	70	**13.73**	−2.322	90.03	0.018 *	0.42
	4	15.82 (4.77)	65	17.13 (3.35)	70	**8.52**	−1.837	113.92	0.07	0.32
	5	16.76 (4.88)	62	17.89 (3.29)	70	**13.44**	−1.536	104.96	0.12	0.27
Daily communication activities in educational settings	3	9.94 (2.82)	62	10.87 (1.52)	70	**20.43**	−2.329	91.14	0.017 *	0.42
	4	10.06 (2.87)	65	11.29 (1.94)	70	11.72	−2.887	111.16	0.004 **	0.50
	5	10.90 (3.09)	62	11.90 (1.37)	70	**55.79**	−2.345	82.05	0.016 *	0.43

Note: * *p* < 0.05. ** *p* < 0.01. CI: cochlear implant; NH: normal-hearing. In cases where the assumption of homogeneity of variances was not met (as evidenced by a significant Levene’s test), Welch’s *t*-test was applied instead of the standard Student’s *t*-test. The corresponding values in the results table are shown in bold. *p*-values were corrected for multiple comparisons using the Bonferroni procedure. SESR = Composite index of family socioeconomic resources. This composite variable combines measures of family economic level, parental education, and the number of books in the home.

**Table 3:** Correlational analysis of language abilities and variables in educational settings for preschool children with cochlear implants (n = 189) (Format adapted from He & Chung, 2026 [46]).

	1	2	3	4	5	6	7	8
1. Language abilities	1							
2. Age	0.355 **	1						
3. Age of implantation	−0.211 **	0.253 **	1					
4. Composite index (SESR)	0.268 **	−0.035	−0.231 **	1				
5. Auditory utilization status	0.631 **	0.062	−0.235 **	0.202 **	1			
6. Learning attentional state	0.516 **	0.182 *	−0.13	0.191 **	0.569 **	1		
7. Language learning activities in educational settings	0.683 **	0.147 *	−0.123	0.190 **	0.624 **	0.547 **	1	
8. Daily communication activities in educational settings	0.642 **	0.134	−0.198 **	0.216 **	0.666 **	0.500 **	0.745 **	1

Note: * *p* < 0.05. ** *p* < 0.01. SESR = Composite index of family socioeconomic resources. This composite variable combines measures of family economic level, parental education, and the number of books in the home.

**Table 4:** Correlational analysis of language abilities and variables in educational settings for normal-hearing children (n = 210) (Format adapted from He & Chung, 2026 [46]).

	1	2	3	4	5	6
1. Language abilities	1					
2. Age	0.735 **	1				
3. Composite index (SESR)	0.116	0.031	1			
4. Learning attentional state	0.387 **	0.235 **	0.191 **	1		
5. Language learning activities in educational settings	0.378 **	0.169 *	0.200 **	0.473 **	1	
6. Daily communication activities in educational settings	0.377 **	0.251 **	0.118	0.450 **	0.555 **	1

Note: * *p* < 0.05. ** *p* < 0.01. SESR = Composite index of family socioeconomic resources. This composite variable combines measures of family economic level, parental education, and the number of books in the home.

**Table 5:** A hierarchical regression model for predicting language abilities of preschool children with cochlear implants within educational settings (n = 189). (Format adapted from He & Chung, 2026 [46]).

		Unstandardized Coefficient		Standardized Coefficient	t	*p*	VIF	R^2^	Adjusted R^2^	△R^2^	F
Model		β	Standard Error	Beta							
1	(Constant)	32.34	18.27	−	1.77	0.078	−	0.223	0.214	0.223	26.658 **
	Age	29.43	4.51	0.44	6.53	<0.001	1.07				
	Age of implantation	−21.071	4.38	−0.322	−4.814	<0.001	1.07				
2	(Constant)	−4.718	20.82	−	−0.227	0.821	−	0.269	0.257	0.046	22.676 **
	Age	29.05	4.39	0.43	6.63	<0.001	1.07				
	Age of implantation	−17.634	4.38	−0.269	−4.031	<0.001	1.13				
	Composite index (SESR)	2.57	0.75	0.22	3.41	0.001	1.06				
3	(Constant)	−127.309	20.29	−	−6.275	<0.001	−	0.541	0.531	0.272	54.177 **
	Age	24.41	3.51	0.36	6.95	<0.001	1.09				
	Age of implantation	−9.351	3.57	−0.143	−2.622	0.009	1.19				
	Composite index (SESR)	1.60	0.61	0.14	2.63	0.009	1.08				
	Auditory utilization status	11.37	1.09	0.55	10.44	<0.001	1.10				
4	(Constant)	−135.178	20.31	−	−6.656	< 0.001	−	0.555	0.542	0.014	45.558 **
	Age	22.86	3.53	0.34	6.48	<0.001	1.13				
	Age of implantation	−9.114	3.52	−0.139	−2.587	0.010	1.19				
	Composite index (SESR)	1.46	0.60	0.13	2.42	0.016	1.09				
	Auditory utilization status	9.75	1.28	0.47	7.65	<0.001	1.55				
	Learning attentional state	3.57	1.50	0.15	2.37	0.019	1.54				
5	(Constant)	−132.919	19.26	−	−6.903	<0.001	−	0.602	0.589	0.047	45.868 **
	Age	21.06	3.37	0.31	6.25	<0.001	1.14				
	Age of implantation	−7.991	3.35	−0.122	−2.387	0.018	1.19				
	Composite index (SESR)	1.23	0.57	0.11	2.14	0.034	1.10				
	Auditory utilization status	6.34	1.41	0.31	4.48	<0.001	2.12				
	Learning attentional state	2.43	1.45	0.10	1.68	0.094	1.59				
	Daily communication activities in educational settings	5.61	1.21	0.30	4.66	<0.001	1.92				
6	(Constant)	−124.832	18.24	−	−6.843	<0.001	−	0.648	0.634	0.046	47.530 **
	Age	20.60	3.18	0.31	6.48	<0.001	1.14				
	Age of implantation	−9.005	3.17	−0.137	−2.845	0.005	1.20				
	Composite index (SESR)	1.16	0.54	0.10	2.15	0.033	1.10				
	Auditory utilization status	5.18	1.36	0.25	3.82	<0.001	2.19				
	Learning attentional state	0.94	1.40	0.04	0.67	0.504	1.67				
	Daily communication activities in educational settings	2.11	1.35	0.11	1.57	0.119	2.69				
	Language learning activities in educational settings	3.85	0.80	0.34	4.85	<0.001	2.56				

Note: ** *p* < 0.01. SESR = Composite index of family socioeconomic resources. This composite variable combines measures of family economic level, parental education, and the number of books in the home.

**Table 6:** A hierarchical regression model for predicting language abilities of normal-hearing preschool children within educational settings (n = 210). (Format adapted from He & Chung, 2026 [46]).

		Unstandardized Coefficient		Standardized Coefficient	t	*p*	VIF	R^2^	Adjusted R^2^	△R^2^	F
Model		β	Standard Error	Beta							
1	(Constant)	40.54	7.93	−	5.11	<0.001	−	0.541	0.538	0.541	244.753 **
	Age	30.38	1.94	0.74	15.65	<0.001	1.00				
2	(Constant)	31.15	9.17	−	3.40	0.001	−	0.549	0.545	0.009	126.118 **
	Age	30.26	1.93	0.73	15.69	<0.001	1.00				
	Composite index (SESR)	0.80	0.40	0.09	2.00	0.047	1.00				
3	(Constant)	9.19	9.95	−	0.92	0.357	−	0.592	0.586	0.043	99.575 **
	Age	28.21	1.89	0.68	14.91	<0.001	1.06				
	Composite index (SESR)	0.46	0.39	0.05	1.18	0.241	1.04				
	Learning attentional state	3.41	0.74	0.22	4.64	<0.001	1.10				
4	(Constant)	−8.69	11.90	−	−0.73	0.466	−	0.605	0.598	0.014	78.617 **
	Age	27.37	1.89	0.66	14.46	<0.001	1.09				
	Composite index (SESR)	0.42	0.39	0.05	1.09	0.278	1.04				
	Learning attentional state	2.57	0.79	0.16	3.24	0.001	1.31				
	Daily communication activities in educational settings	2.67	1.01	0.13	2.65	0.009	1.29				
5	(Constant)	−15.02	11.83	−	−1.27	0.206	−	0.624	0.614	0.018	67.568 **
	Age	27.39	1.85	0.66	14.78	<0.001	1.09				
	Composite index (SESR)	0.27	0.38	0.03	0.71	0.476	1.06				
	Learning attentional state	1.85	0.81	0.12	2.29	0.023	1.42				
	Daily communication activities in educational settings	1.20	1.09	0.06	1.10	0.273	1.58				
	Language learning activities in educational settings	1.85	0.59	0.17	3.14	0.002	1.61				

Note: ** *p* < 0.01. SESR = Composite index of family socioeconomic resources. This composite variable combines measures of family economic level, parental education, and the number of books in the home.

## Data Availability

The data supporting the findings of this study are available from the corresponding author upon request.

## Supplementary Materials

The following supporting information can be downloaded at: https://www.mdpi.com/article/10.3390/children13060806/s1, File S1: Survey questionnaire on the current status of language activities in the educational settings of preschool children with cochlear implants.

## Abbreviations

The following abbreviations are used in this manuscript:

CIs	Cochlear implants
NH	Normal-hearing
SESR	Socioeconomic status resource
ASQ	Ages & Stages Questionnaire
MCELP-CS	Mandarin Clinical Evaluation of Language for Preschoolers’ Core Scale
