# Supplementary material for: Language Learning Activities in Educational Settings Incrementally Predict Language Ability Beyond Daily Conversation in Preschoolers with Cochlear Implants: A Comparative Study with Normal-Hearing Peers
Source: Children (Basel). 2026 Jun 11;13(6):806. doi: 10.3390/children13060806 (PMC13298334; doi:10.3390/children13060806)
Supplement: Supplementary file 1 [file children-13-00806-s001.zip › children-4315823-supplementary.pdf]

Supplementary Material File S1

Survey questionnaire on the current status of language activities in educational settings of preschool children with cochlear implants

I . Basic information of preschoolers with cochlear implants

1. Age:______ years_____ months
2. Gender: 1) Male 2) Female
3. Age at hearing loss diagnosis:______ years______ months
4. Age at cochlear implant initiation: ______years______ months

5. Cochlear implant status
   1) Bilateral 2) Right Ear 3) Left Ear
   If implanted in only one ear, does the other ear use a hearing aid?
   1)Yes 2) No

6. Hearing levels
   ① Average pre-implant hearing (Unassisted)
   1) Right ear_________dB HL 2) Left ear_________dB HL
   ② Average hearing level after cochlear implantation or hearing aid use
   1) Right ear_________dB HL 2) Left ear_________dB HL
7. Does your child have any other disabilities besides hearing impairment?
   1) Yes 2) No

8. Educational attainment of your child's parents
   ① Father's educational attainment
   1) Junior high school or below 2) High school 3) College diploma
   4) Bachelor's degree 5) Master's degree or above
   ② Mother's educational attainment
   1) Junior high school or below 2) High school 3) College diploma
   4) Bachelor's degree 5) Master's degree or above

9. Your household's economic level (annual income)
   1) 0-30,000 yuan 2) 30,000-80,000 yuan 3) 80,000-150,000 yuan
   4) 150,000-300,000 yuan 5) 300,000 yuan and above

10. How many books do your children own at home?
    1) Fewer than 10 books 2) 11-30 books 3) 31-60 books 4) 61-100 books 5) More than 100 books

11. How many books do the adults in your household own?
    1) Less than 10 books 2) 11-30 books 3) 31-60 books 4) 61-100 books 5) Over 100 books

Ⅱ. Auditory utilization status and learning attentional states in educational settings

NO.	Item	Poor	Not very good	Average	good	Very good
1	How does this child perceive sounds?	1	2	3	4	5
2	How is this child's ability to distinguish between different sounds in language?	1	2	3	4	5
3	Did the child seem confused or hesitant in response to the teacher’s verbal instructions?	1	2	3	4	5

NO.	Item	Very low/<br>Very short/<br>Rarely	A bit low/<br>A bit short/<br>Occasionally	Average	A bit tall/<br>A bit long/<br>Often	Very high/<br>Very long/<br>Always
1	How easily is this child distracted? For example: Is he or she easily distracted by external factors?	1	2	3	4	5
2	How long can this child concentrate? For example: how long can they focus on a single task?	1	2	3	4	5
3	How does this child behave when listening attentively to a teacher or friend?	1	2	3	4	5

III. Language learning activities in educational settings

NO.	Item	Rarely	Occasionally	Sometimes	Often	Always
1	Does this child actively participate in conversation activities? For example: morning discussions, sharing after playtime, such as “My Toys” or “My Family”	1	2	3	4	5
2	Does this child actively participate in storytelling activities?For example: picture book storytelling, describing pictures, role-playing, etc.	1	2	3	4	5
3	Does this child actively participate in listening and speaking games? For example: games involving sounds, vocabulary, sentence expansion, and rhythm; word chains, riddles, etc.	1	2	3	4	5
4	Does this child actively participate in activities related to literary works? For example: learning nursery rhymes, poetry, and prose	1	2	3	4	5
5	Does this child actively participate in early reading activities? For example: participating in picture book reading sessions at the kindergarten’s book corner	1	2	3	4	5
6	To what extent does this child interact with other children?	1	2	3	4	5
7	To what extent does this child interact with teachers or other staff members at the preschool?	1	2	3	4	5
8	Is this child able to clearly express his or her needs verbally?	1	2	3	4	5
